# Biological Ingredient Analysis of Traditional Herbal Patent Medicine Fuke Desheng Wan Using the Shotgun Metabarcoding Approach

**DOI:** 10.3389/fphar.2021.607197

**Published:** 2021-08-17

**Authors:** Hongbo Xie, Qing Zhao, Mengmeng Shi, Weijun Kong, Weishan Mu, Baoli Li, Jingyi Zhao, Chunying Zhao, Jing Jia, Jinxin Liu, Linchun Shi

**Affiliations:** ^1^ Hebei Key Laboratory of Study and Exploitation of Chinese Medicine, Chengde Medical University, Chengde, China; ^2^ Department of Pharmacy, Baoding First Central Hospital, Baoding, China; ^3^ Institute of Medicinal Plant Development, Chinese Academy of Medical Sciences, Peking Union Medical College, Beijing, China; ^4^ Department of Pharmacy, Affiliated Hospital of Shandong University of Traditional Chinese Medicine, Jinan, China

**Keywords:** shotgun metabarcoding, traditional herbal patent medicine, biological ingredients, weeds, fungi

## Abstract

With the widespread use of traditional medicine around the world, the safety and efficacy of traditional herbal patent medicine have become an increasing concern to the public. However, it is difficult to supervise the authenticity of herbal materials in mixed herbal products according to the current quality standards, especially for traditional herbal patent medicine, with a distinct variance in the dosage of herbal materials. This study utilized the shotgun metabarcoding approach to analyze the biological ingredients of Fuke Desheng Wan (FKDSW), which is an effective traditional herbal product for the treatment of dysmenorrhea. Six herbal materials were collected, and a lab-made mock FKDSW sample was produced to establish a method for the authentication assessment of biological ingredients in traditional herbal patent medicine based on shotgun metabarcoding. Furthermore, four commercial FKDSW samples were collected to verify the practicality of the shotgun metabarcoding approach. Then, a total of 52.16 Gb raw data for 174 million paired-end reads was generated using the Illumina NovaSeq sequencing platform. Meanwhile, 228, 23, and 14 operational taxonomic units (OTUs) were obtained for the ITS2, *matK*, and *rbcL* regions, respectively, after bioinformatic analysis. Moreover, no differences were evident between the assembly sequences obtained *via* shotgun metabarcoding and their corresponding reference sequences of the same species obtained *via* Sanger sequencing, except for part of the ITS2 and *matK* assembly sequences of *Paeonia lactiflora* Pall., *Saussurea costus* (Falc.) Lipsch*.* and *Bupleurum chinense* DC. with 1–6 different bases. The identification results showed that all six prescribed ingredients were successfully detected and that the non-authentic ingredient of Bupleuri Radix (Chaihu, *Bupleurum chinense* DC. or *Bupleurum scorzonerifolium* Willd.) was found in all the commercial samples, namely *Bupleurum falcatum* L. Here, 25 weed species representing 16 genera of ten families were detected. Moreover, 26 fungal genera belonging to 17 families were found in both lab-made and commercial FKDSW samples. This study demonstrated that the shotgun metabarcoding approach could overcome the biased PCR amplification and authenticate the biological ingredients of traditional herbal patent medicine with a distinct variance in the dosage of the herbal materials. Therefore, this provides an appropriate evaluation method for improving the safety and efficacy of traditional herbal patent medicine.

## Introduction

Dysmenorrhea is a common gynecological disease that can harm the health, work status, and quality of life of women. Research was conducted in various countries to determine the number of adolescents and young women suffering from dysmenorrhea, the results of which indicated that the prevalence rate of this condition ranged from 34% (Egypt) to 94% (Oman) (De Sanctis et al., 2016). Although the prescribed first-line therapy for dysmenorrhea is non-steroidal anti-inflammatory drugs, which usually alleviate menstrual pain by inhibiting peripheral, systemic prostaglandins, and their corresponding downstream effects, there are still approximately 18% of women who continue to suffer from the condition who display a distinct resistance to this treatment (Iacovides et al., 2015; Oladosu et al., 2018). As a result, traditional herbal medicines have been proposed as alternative therapies for dysmenorrhea (Oladosu et al., 2018). Fuke Desheng Wan (FKDSW) has been recommended as a gynecological medicine for curing dysmenorrhea caused by liver depression or the stagnancy of both blood and qi (Commission, 1994). This treatment exhibits a unique curative effect in relieving dysmenorrhea, regulating abnormal menstruation, and improving complications (Commission, 1994). FKDSW is a honeyed pill consisting of six herbal materials, including Angelicae Sinensis Radix (Danggui), Paeoniae Radix Alba (Baishao), Aucklandiae Radix (Muxiang), Notopterygii Rhizoma et Radix (Qianghuo), Leonuri Herba (Yimucao), and Bupleuri Radix (Chaihu), at significantly different and very precise dosages. Leonuri Herba (Yimucao) is the primary ingredient in FKDSW at a level of 59.26%, whereas the content of Aucklandiae Radix (Muxiang), Notopterygii Rhizoma et Radix (Qianghuo), and Bupleuri Radix (Chaihu) displayed the lowest dosage level at 3.70%. The quality control and biological ingredient assessment of FKDSW are challenging compared to other traditional herbal patent medicines with similar dosage levels of each ingredient. In this case, the characteristics of medicinal materials with low content will be overwhelmed by the characteristics of medicinal materials with high content. Although the thin-layer chromatography identification method used for FKDSW, involving Angelicae Sinensis Radix (Danggui) and Paeoniae Radix Alba (Baishao), is recorded in the current standard (Commission, 1994), it is not enough to authenticate all of its labeled ingredients due to the current methods don’t cover all the ingredients. For example, an authenticity survey revealed that approximately 35.3% of Bupleuri Radix (Chaihu) samples in herbal markets were identified to be adulterants in 85 samples of Bupleuri Radix (Chaihu) (Wang et al., 2017). Moreover, there are lots of researches proved that the widespread adulteration of commercial herbal products have been found throughout the global market (van der Valk et al., 2017; Ichim, 2019; Ichim et al., 2020). For example, *Clematis armandii*, an adulterant of Akebiae Caulis (Mutong, *Akebia trifoliata*), was detected in the commercial herbal product of Longdan Xiegan Wan (Xin et al., 2018a). And there are 7% of herbal products of the Lonicerae japonicae Flos contained both of two adulterants Eucommiae Folium and Lonicerae Flos (Gao et al., 2017). Ichim (2019) and Ichim et al. (2020) found that herbal products containing undeclared contaminant, substitute, and filler species, or none of the labeled species were distributed across all continents and regions.

Compared to traditional pharmacopoeial identification methods including macroscopic, microscopic, chemical authentication approaches, DNA-based technology is more universal and accurate and can discriminate different species based on specific DNA fragments or even complete genomic information without the influence of environmental modification or the limitation of experiences, in spite of DNA-based methods do not provide any quantitative nor qualitative information of the active compounds in the herbal materials or the herbal preparations (Chen et al., 2012; Chen et al., 2018; Raclariu et al., 2018; Grazina et al., 2020). The protocol of DNA-based species identification methods like DNA metabarcoding mainly includes three steps: 1) total DNA extraction, 2) PCR amplification of the target DNA regions with universal primers, and 3) the identification and biodiversity assessment of multiple species in complex herbal preparations and products using high-throughput sequencing technology (Taberlet et al., 2012; Coghlan et al., 2015; Arulandhu et al., 2017; Gao et al., 2017; Song et al., 2017). However, the dependence of all these methods on PCR amplification limits their application in identifying the biological ingredients in traditional herbal patent medicine. For example, there are a large number of substances in traditional herbal materials or herbal products, such as polysaccharides, polyphenols, or excipients, which may inhibit PCR amplification and even lead to false-negative PCR results (Schrader et al., 2012). Furthermore, PCR bias, due to the differential binding of PCR primers to DNA templates, may lead to the loss of target sequences of some taxa or introduce chimeric sequences and other errors (Berry et al., 2011; Porter and Hajibabaei, 2018).

Shotgun metagenomics is capable of untargeted sequencing of all biological genomes from a single bulk sample without PCR amplification (Quince et al., 2017), which has been recognized as an unbiased method for investigating multiple species in various environmental or clinical samples (Bovo et al., 2018; Vijayvargiya et al., 2019; Ye et al., 2019). For example, Bovo et al. (2018) analyzed the environmental DNA samples of honey bees using shotgun metagenomic sequencing and reported five major biological groups in the two samples, including arthropods, plants, fungi, bacteria, and viruses, indicating that this method could be applied in large-scale experiments. Moreover, shotgun metagenomic sequencing holds additional benefits, such as species detection, the discovery of novel species, exploring the potential functions and relative abundance of the species of an organism, while reducing the reliance on independent cultures and PCR amplification approaches (Jovel et al., 2016; Ranjan et al., 2016; Porter and Hajibabaei, 2018; Ye et al., 2019). Although shotgun metagenomic sequencing has the potential of becoming a powerful analytical tool and has been applied to monitor the biological ingredients of traditional herbal patent medicine (Xin et al., 2018a), it presents two inherent difficulties that need to be overcome, namely the considerable amount of data that is generated and the limited number of whole genomic reference sequences. Since the purpose of the traditional herbal patent medicine identification is only to determine the species composition of biological ingredients, selecting the phylogenetic or DNA barcoding markers may provide an alternative, fast, accurate, and lightweight approach (Segata et al., 2012; Sunagawa et al., 2013). With the development of high-throughput sequencing and bioinformatics, shotgun metabarcoding is expected to become a highly effective regulatory approach for the authentication assessment of biological ingredients in herbal product mixtures.

FKDSW is selected as the research object in this study. Shotgun metagenomic sequencing is used in conjunction with DNA barcoding (defined as “shotgun metabarcoding” in the present research) to determine the species composition in FKDSW and discuss the feasibility of this approach for monitoring the biological ingredients in traditional herbal patent medicine with distinct variance in the dosage of herbal materials. Meanwhile, this study combines multiple materials evidence to make its strategies and techniques for clearly and efficiently authenticating the herbal materials of FKDSW, effectively supplementing traditional identification methods, which will be useful for improving the holistic quality control of traditional herbal patent medicine.

## Materials and Methods

### Sample Collection

**FIGURE 1:**
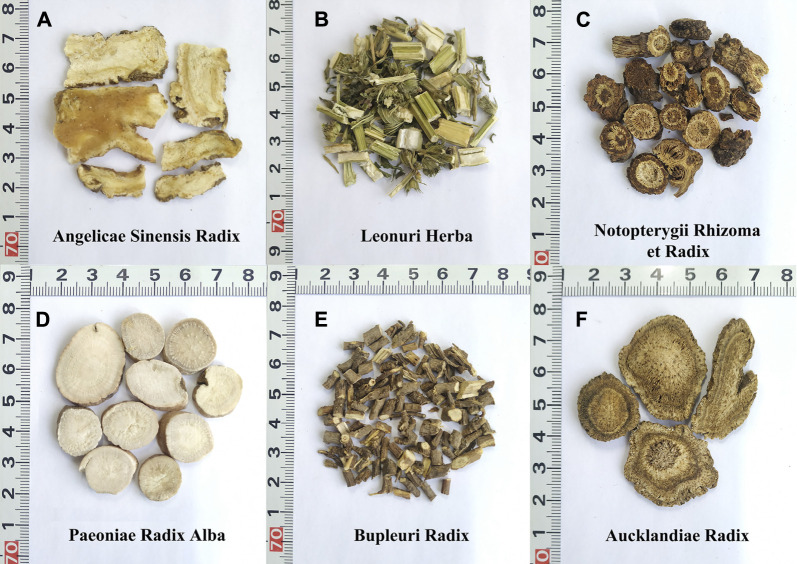
The morphological characteristics of the six herbal materials in FKDSW. **(A)** Angelicae Sinensis Radix (Danggui). **(B)** Leonuri Herba (Yimucao). **(C)** Notopterygii Rhizoma et Radix (Qianghuo). **(D)** Paeoniae Radix Alba (Baishao). **(E)** Bupleuri Radix (Chaihu). **(F)** Aucklandiae Radix (Muxiang).

Six herbal materials were purchased from the Beijing Tongrentang herbal store to prepare the mock FKDSW sample and included Angelicae Sinensis Radix (Danggui, HSYC 2002), Paeoniae Radix Alba (Baishao, HSYC 2036), Aucklandiae Radix (Muxiang, HSYC 2037), Notopterygii Rhizoma et Radix (Qianghuo, HSYC 2038), Leonuri Herba (Yimucao, HSYC 2039), and Bupleuri Radix (Chaihu, HSYC 2078). Then, these materials were authenticated *via* morphological and DNA barcoding identification. In particular, the morphological identification was based on the herbal materials of form, shape and size, color, external markings and texture, organoleptic characters (odor, taste, and mouthfeel) recorded in the Chinese Pharmacopoeia (Commission, 2020). And these materials are deposited in the Institute of Medicinal Plant Development herbarium (herbarium code “IMD”, NYBG: https://www.nybg.org/). The six medicinal materials and their prescribed proportions are shown in Figure 1, Table 1 and Supplementary Table S1. To verify the feasibility and accuracy of the shotgun metabarcoding approach, the mock FKDSW sample was handcrafted in the laboratory according to the preparation method recorded in the pharmaceutical standard (Volume 9, WS3-B-1743-94) (Commission, 1994), and named HSZY167. Four batches of commercial FKDSW samples were purchased from drug stores to test the applicability of shotgun metabarcoding and numbered as HSZY003, HSZY140, HSZY153, and HSZY154.

**TABLE 1:** The prescribed proportion of each herbal ingredient that was labeled in FKDSW.

Herbal material Latin name (pinyin name)	Prescribed proportion (%)
Angelicae Sinensis Radix (Danggui)	14.81
Paeoniae Radix Alba (Baishao)	14.81
Aucklandiae Radix (Muxiang)	3.70
Notopterygii Rhizoma et Radix (Qianghuo)	3.70
Leonuri Herba (Yimucao)	59.26
Bupleuri Radix (Chaihu)	3.70

### DNA Extraction and Quantification

The DNA was extracted from the herbal materials following the previously published DNA barcoding protocol (Liu et al., 2017) and principles for traditional Chinese herbal medicine (Chen et al., 2014). Detailed steps were carried out according to the protocol supplied by the manufacturer of the plant genomic DNA extraction kit [Tiangen Biochemical Technology (Beijing) Co., Ltd, China]. Then, the extracted DNA was amplified *via* PCR with primer sets of ITS2, *matK*, and *rbcL* regions using 2 × Taq master mix (AidLab Biotechnologies Co., Ltd., China). The primer selection and PCR conditions adhered to the barcode of traditional Chinese herbal medicine data system (TCM-BOL) (Chen et al., 2014), the consortium for the barcode of life (CBOL) plant working group (Group, 2009), and the barcode of life data system (BOLD) (Ratnasingham and Hebert, 2007). Then the PCR products were electrophoresed on 1.0% agarose gel using GelRed nucleic acid gel stain (Biotium Biotechnologies Co., Ltd., United States) to determine the integrity.

The metagenomic DNA was extracted from the traditional herbal patent medicine samples according to a method reported (Cheng et al., 2015), with some minor modifications during the pretreatment steps (Xin et al., 2018a). Finally, the concentration and quality of the DNA in all the samples of the herbal materials and traditional herbal patent medicine were determined using a NanoDrop ONE ultra-micro spectrophotometer (Thermo Fisher Scientific, Inc., United States).

### Sequencing, Bioinformatic Analysis, and Species Identification

The PCR products of ITS2, *matK*, and *rbcL* were bi-directionally sequenced using an ABI 3730xL DNA Analyzer (Thermofisher Co., Ltd., United States). Then, the sequencing output files generated *via* Sanger sequencing were aligned and assembled using Codoncode aligner v 9.0.1 (CodonCode Corp., Dedham, MA, United States).

The Illumina NovaSeq platform was used for the shotgun sequencing of the traditional herbal patent medicine samples after a meta-genomic DNA paired-end library was constructed with sheared fragments. The sequencing output files generated by high-throughput sequencing were used to remove the adapters, and low quality reads using Trimmonmatic v0.38 (Bolger et al., 2014). The ITS2, *matK*, and *rbcL* sequences were downloaded from the National Center for Biotechnology Information (NCBI) to construct the reference database. And the reads after quality control were aligned to the reference database using basic local alignment search tool (BLAST), then the targeted paired-end reads of ITS2, *matK*, and *rbcL* were enriched by local python scripts (Shi et al., 2019). The enriched reads were assembled with MEGAHIT v1.2.9 and MetaSPAdes v3.13.2 (Li et al., 2015; Nurk et al., 2017), while the k-mer values ranged from 31 to 127. The assembled contigs were clustered, and redundant data were removed using cd-hit with 100% identity (Li and Godzik, 2006). Cutadapt v2.10 was employed to remove the primer sequences of the conventional DNA barcoding *matK* and *rbcL* regions (Martin, 2011), while the ITS2 region was annotated using the hidden Markov model (HMM)-based annotation method (Keller et al., 2009). Chimeric sequences were removed by UCHIME v4.2 (Edgar et al., 2011). The remaining sequences were clustered into operational taxonomic units (OTUs) at 100% sequence similarity using Usearch v11 (https://www.drive5.com/usearch/). The OTUs of the representative sequences were further processed with Bowtie2 v2.4.1 and samtools v1.10, where the former was used to map the shotgun paired-end reads to the representative sequences, and the latter was employed to calculate the sequencing depth and coverage values (Langmead and Salzberg, 2012; Etherington et al., 2015). Then, the OTUs were manually removed, satisfying the following parameters: sequencing depth ≤3 and/or coverage ≤95%. The BLAST search (Camacho et al., 2009) was applied according to the information in the GenBank (Benson et al., 2014), TCM-BOL (Chen et al., 2014), and BOLD (Ratnasingham and Hebert, 2007) databases to obtain the remaining OTUs and facilitate species identification. All sequences acquired *via* Sanger sequencing were deposited in the GenBank database. The GenBank accession numbers are shown in Table 2. Finally, the resultant BLAST files were imported into MEGAN v6.18.9 for taxonomic analysis and to obtain the statistics regarding the species composition of the traditional herbal patent medicine (Huson et al., 2016).

**TABLE 2:** The species of herbal materials assigned by classic DNA barcoding and their GenBank accession numbers.

Sample ID	Species	GenBank accession number		
		ITS2	*matK*	*rbcL*
HSYC2002	*Angelica sinensis* (Oliv.) Diels	MN712234	MN746764	MN729559
HSYC2036	*Paeonia lactiflora* Pall.	MN712235	—	—
HSYC2037	*Saussurea costus* (Falc.) Lipsch.	MN712236	MW000342	MW000336
HSYC2038	*Hansenia weberbaueriana* (Fedde ex H.Wolff) Pimenov & Kljuykov	MN712237	—	—
HSYC2039	*Leonurus japonicus* Houtt.	MN712238	MW000343	MW000337
HSYC2078	*Bupleurum chinense* DC.	MN712239	MW000344	—

Note: “—” indicates that the corresponding reference sequence was not obtained.

## Results

### The Authentication of the Six Herbal Materials in FKDSW and Their Reference ITS2, *matK*, and *rbcL* DNA Barcodes

The authentication of the six herbal materials was further verified *via* DNA barcoding to ensure their accuracy. All the ITS2 sequences of the six herbal ingredients used for producing the lab-made mock FKDSW sample were successfully obtained and assigned to species by blasting to the TCM-BOL database. The *matK* sequences of Paeoniae Radix Alba (Baishao) and Notopterygii Rhizoma et Radix (Qianghuo), and the *rbcL* sequences of Paeoniae Radix Alba (Baishao), Notopterygii Rhizoma et Radix (Qianghuo), and Bupleuri Radix (Chaihu) failed to be amplified by the universal primers, while only the *matK* and *rbcL* sequences of the remaining herbal materials were used for species assignment by blasting to the BOLD and GenBank nt databases. The comprehensive species identification results of three DNA barcodes showed that the six herbal materials were authentic species recorded in the Chinese Pharmacopoeia, which were consistent with the morphological identification results. Finally, the original species of Angelicae Sinensis Radix (Danggui), Paeoniae Radix Alba (Baishao), Aucklandiae Radix (Muxiang), Notopterygii Rhizoma et Radix (Qianghuo), Leonuri Herba (Yimucao), and Bupleuri Radix (Chaihu) were verified to be *Angelica sinensis* (Oliv.) Diels, *Paeonia lactiflora* Pall., *Saussurea costus* (Falc.) Lipsch*.* (synonym: *Aucklandia costus* Falc.), *Hansenia weberbaueriana* (Fedde ex H.Wolff) Pimenov & Kljuykov, *Leonurus japonicus* Houtt., and *Bupleurum chinense* DC. The quantity and quality of the extracted DNA are shown in Supplementary Table S2. The identification results and GenBank accession numbers are presented in Table 2.

### An Overview of the High-Throughput Sequencing Data and Shotgun Metabarcoding Assembly

The average extracted DNA concentration from the five FKDSW samples was 110.5 ng/μL, while the A_260_/A_280_ values ranged from 1.8 to 1.9, indicating that the quantity and quality of the metagenomic DNA extracted from FKDSW adequately met the requirements of high throughput sequencing (Supplementary Table S3). A total of 52.16 Gb of clean data was generated from about 174 million paired-end reads using the Illumina NovaSeq sequencing platform. On average, 10.43 Gb of clean data was acquired for each FKDSW sample. The commercial HSZY003 sample displayed the largest quantity of sequencing data at 18.14 Gb, while HSZY140 had the least at 6.6 Gb. Furthermore, 371,553 paired-end reads were enriched for ITS2, *matK*, and *rbcL* from the clean data, while the number of paired-end reads belonging to the ITS2, *matK*, and *rbcL* regions was 217,085, 52,642, and 101,826, respectively. Further analysis showed that the number of reads enriched for each marker was consistent with the clean data of its corresponding sample (Supplementary Table S4). In total, 1,248 unique contigs were generated, in which the number of unique contigs of ITS2, *matK*, and *rbcL* was 888, 150, and 210, respectively. Moreover, 466 DNA barcodes were obtained from ITS2, *matK*, and *rbcL* after conventional DNA barcoding region annotation and chimera detection. Subsequently, these DNA barcodes in the conventional DNA barcoding region were clustered into 265 OTUs comprising 228, 23, and 14 OTUs for ITS2, *matK*, and *rbcL*, respectively (Table 3). Considering the potential sequencing or assembly errors, further accuracy verification was performed for each OTU *via* read mapping. Finally, 133 reliable OTUs were obtained, including 98 ITS2 OTUs, 21 *matK* OTUs, and 14 *rbcL* OTUs.

**TABLE 3:** The assembly results of three DNA barcodes in the FKDSW samples.

	ITS2	*matK*	*rbcL*
Number of unique contigs	888	150	210
Number of DNA barcodes after annotation and chimera detection	386	47	33
Number of OTUs	228	23	14
Average length (bp)	210.5	844.2	703
GC content (%)	59	33.2	42.8

### The Species Detection of in the Lab-Made Mock Sample and the Verification of the Assembly Results

A total of 58 reliable OTUs were obtained from the ITS2, *matK*, and *rbcL* regions in the lab-made mock FKDSW sample, including 36 ITS2 OTUs, 12 *matK* OTUs, and 10 *rbcL* OTUs. The species assignment results showed that all the original species of the six prescribed herbal ingredients were successfully detected and included *Angelica sinensis*, *Paeonia lactiflora*, *Saussurea costus*, *Hansenia weberbaueriana*, *Leonurus japonicas,* and *Bupleurum chinense* (Table 4). Of these OTUs in the ITS2, *matK*, and *rbcL* regions, seven were identified as *Bupleurum chinense*, which exhibits the most significant number of OTUs among the six labeled species. Although there are only three OTUs belonging to *Leonurus japonicas*, which contains the largest number of mapping reads. And 4,528, 9,906, and 10,263 reads could be mapped to the ITS2, *matK*, and *rbcL* OTUs of *Leonurus japonicas*, respectively.

**TABLE 4:** Species detection of the ITS2, *matK*, and *rbcL* DNA barcoding regions obtained *via* shotgun metabarcoding in the lab-made mock FKDSW sample.

Herbal ingredients	Original species	ITS2	*matK*	*rbcL*
Angelicae Sinensis Radix (Danggui)	*Angelica sinensis*	√	√	√
Paeoniae Radix Alba (Baishao)	*Paeonia lactiflora*	√	√	√
Aucklandiae Radix (Muxiang)	*Saussurea costus*	√	√	√
Notopterygii Rhizoma et Radix (Qianghuo)	*Hansenia weberbaueriana*	√	√	√
Leonuri Herba (Yimucao)	*Leonurus japonicus*	√	√	√
Bupleuri Radix (Chaihu)	*Bupleurum chinense*	√	√	√

Note: “√” means that the assembly sequence of this species was obtained.

To determine the assembly accuracy of ITS2, *matK*, and *rbcL* from the shotgun sequencing data, this study compared these sequences (called “assembly sequences” here) with their corresponding reference sequences of the same species obtained *via* Sanger sequencing. For the ITS2 region, all assembly sequences and reference sequences of the original species belonging to six prescribed herbal ingredients were obtained *via* the two sequencing methods, respectively (Supplementary Figure S1). The assembly sequences obtained from *Angelica sinensis*, *Hansenia weberbaueriana*, and *Leonurus japonicas* were absolutely consistent with the reference sequences obtained *via* Sanger sequencing. Three types of ITS2 assembly sequences were evident for *Paeonia lactiflora*, one of which was consistent with the reference sequence, while the others were 1–2 base substitutions for the reference sequences. Two types of haplotypes of ITS2 assembly sequences were obtained from *Saussurea costus* and *Bupleurum chinense*, respectively, with 0–4 base difference from the reference sequences. For the *matK* sequences, four species shared sequences that were generated *via* the two sequencing methods. The assembly sequences from the *matK* region of *Angelica sinensis*, *Saussurea costus,* and *Leonurus japonicas* were identical to the reference sequences. As for the three *matK* assembly sequences of *Bupleurum chinense*, there were 0, 1, and 6 base differences in these assembly sequences compared with its reference sequences. The assembly sequences obtained *via* shotgun metabarcoding were identical to their corresponding reference sequences of the same species obtained *via* Sanger sequencing for *Angelica sinensis*, *Saussurea costus,* and *Leonurus japonicas* in the *rbcL* region.

### The Authentic and Non-Authentic Ingredients Detected in the Commercial Samples

**FIGURE 2:**
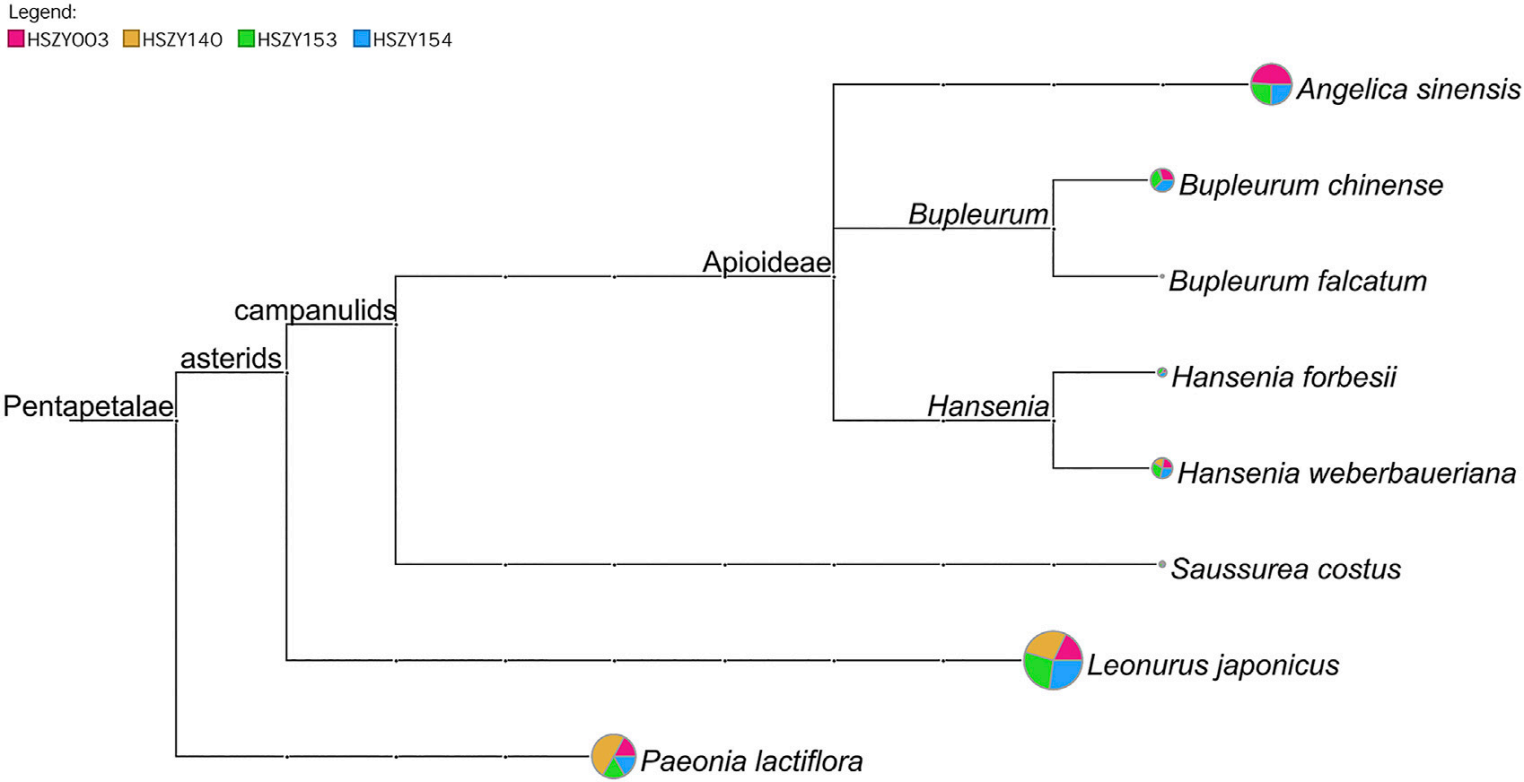
A comparison of the taxonomic analytical results of the prescribed biological ingredients in four commercial FKDSW samples based on ITS2 sequences. Each taxonomic node represents a taxon in the NCBI taxonomy and is labeled according to its name. The pie chart shows the ratio of shotgun reads assigned to the corresponding taxon in four samples, while the size of the circle is scaled logarithmically to indicate the number of shotgun reads assigned to the taxon.

In total, 98 reliable ITS2 OTUs were obtained from the commercial samples, of which 11 OTUs were identified as the original plant species of six prescribed herbal ingredients. The BLAST results demonstrated that *Angelica sinensis*, *Paeonia lactiflora*, *Saussurea costus*, *Hansenia weberbaueriana*, *Leonurus japonicus,* and *Bupleurum chinense* were detected based on the ITS2 sequences in four commercial samples. *Hansenia forbesii* (H.Boissieu) Pimenov & Kljuykov, which is another botanical source species for Notopterygii Rhizoma et Radix (Qianghuo), was also detected in the HSZY003, HSZY153, and HSZY154 samples. In addition, *Bupleurum falcatum* L. was detected in four commercial samples and is a non-authentic ingredient for Bupleuri Radix (Chaihu), as shown in Figure 2 and Supplementary Table S5.

Among the obtained 21 *matK* OTUs and 14 *rbcL* OTUs, *Paeonia lactiflora* and *Leonurus japonicus* were detected in the four commercial FKDSW samples. The original plant species of Angelicae Sinensis Radix (Danggui) and Bupleuri Radix (Chaihu) were determined at the genus level in the commercial FKDSW samples. For example, one OTU obtained from the *matK* region was identified as several species belonging to the genus *Angelica* in the four commercial FKDSW samples, but could not be assigned to a specific species. Moreover, five OTUs obtained from the *matK* and *rbcL* regions belonged to *Bupleurum* in the three commercial FKDSW samples, of which three were shared by HSZY003, HSZY153, and HSZY154. In addition, there are other contaminating species were found in the commercial FKDSW samples, which are described in detail in Supplementary Figures S2, S3, and Supplementary Table S5.

In general, six prescribed ingredients in the four commercial FKDSW samples were successfully detected with the combination of the ITS2, *matK*, and *rbcL* sequences. The ITS2 region displayed higher identification efficiency at the species level, while it was difficult to determine some Asteraceae and Apiaceae at the species level using the *matK* and *rbcL* sequences.

### Unlabeled Plant Species Detected in the Lab-Made Mock and Commercial Samples

The contaminating plant species found in this study were represented by 68 OTUs from the ITS2, *matK*, and *rbcL* regions, and were classified into 14 families that included 24 genera and 30 species, while the remainder could be resolved at the genera or family levels, as shown in Supplementary Tables S6–S8. Ten plant families with 16 genera that included 25 species denoted common field weeds, such as *Artemisia annua* L., *Artemisia argyi* H.Lév. & Vaniot, *Artemisia scoparia* Waldst. & Kit., and *Erigeron canadensis* L. from Asteraceae, *Humulus scandens* and *Cannabis sativa* L. from Cannabaceae, *Ipomoea purpurea* (L.) Roth and *Ipomoea nil* (L.) Roth from Convolvulaceae, *Setaria viridis* (L.) P.Beauv. from Poaceae, as well as *Abutilon theophrasti* Medik. and *Hibiscus trionum* L. from Malvaceae. Of these, *Artemisia*, *Humulus,* and *Ipomoea* signified the three most common plant genera of these potential species. For the ITS2 region, a total of 11 families with 22 genera that included 28 species were identified for the contaminating plant species. Additionally, six families with six genera that included six species, as well as four families with four genera that included three species, were detected for the *matK* and *rbcL* regions, respectively. Furthermore, there were distinct differences in the relative species abundance of the contaminating plant species between the mock and four commercial FKDSW samples. For example, only four genera of botanical contamination were found in the mock FKDSW sample, including *Artemisia*, *Humulus*, *Ipomoea,* and *Erigeron*. However, all botanically contaminating species found in this study were detected in the commercial samples, containing a small number of other arbor species that included *Ulmus* of Ulmaceae, *Populus* of Salicaceae, *Robinia* of Fabaceae, and *Broussonetia* of Moraceae. The HSZY003 sample comprised a large proportion of botanical contamination, including 23 genera with 29 species.

### The Fungal Communities in the Lab-Made Mock and Commercial Samples

**FIGURE 3:**
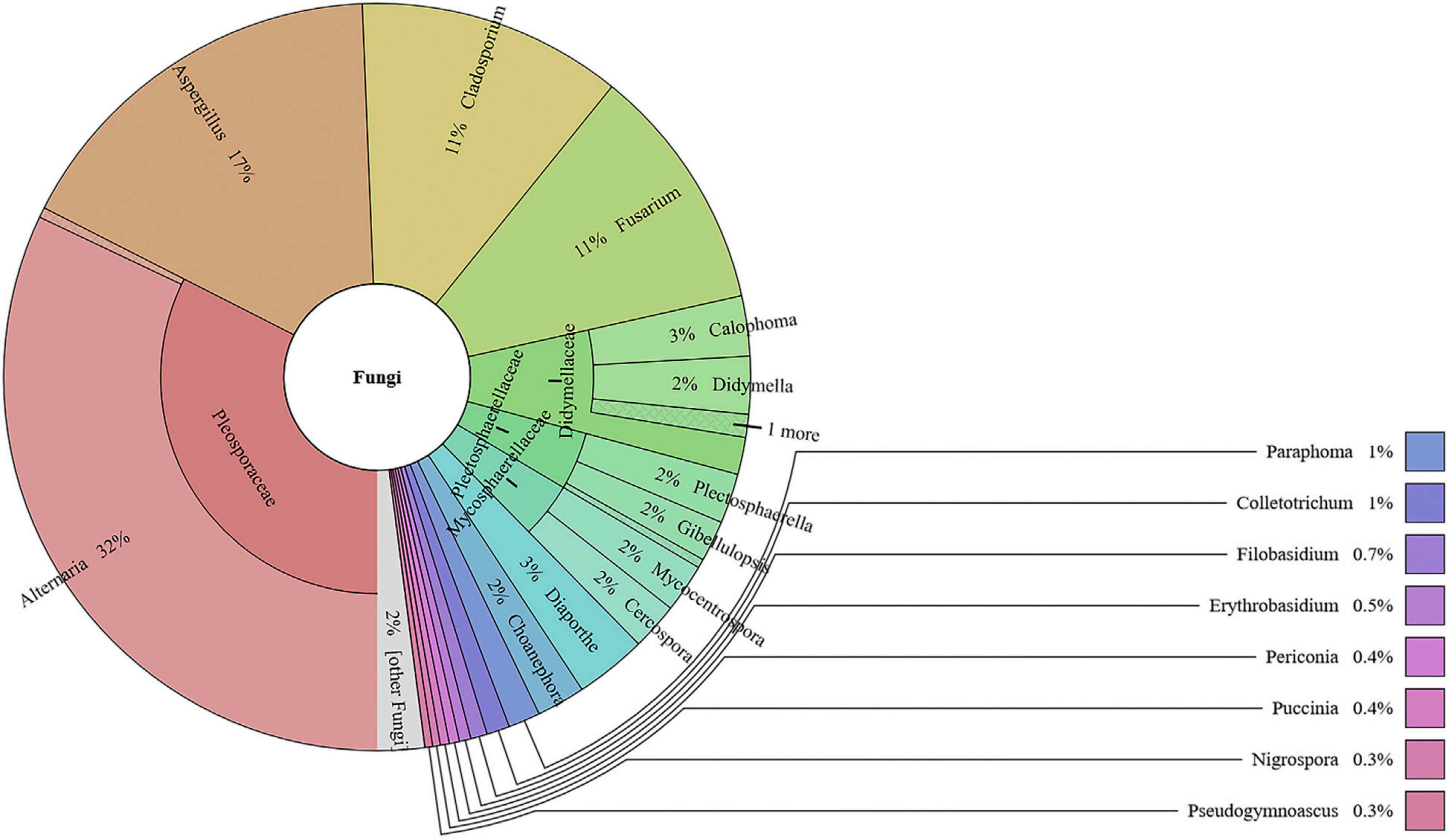
The fungal composition detected in lab-made mock and commercial FKDSW samples at the family and genus levels.

**FIGURE 4:**
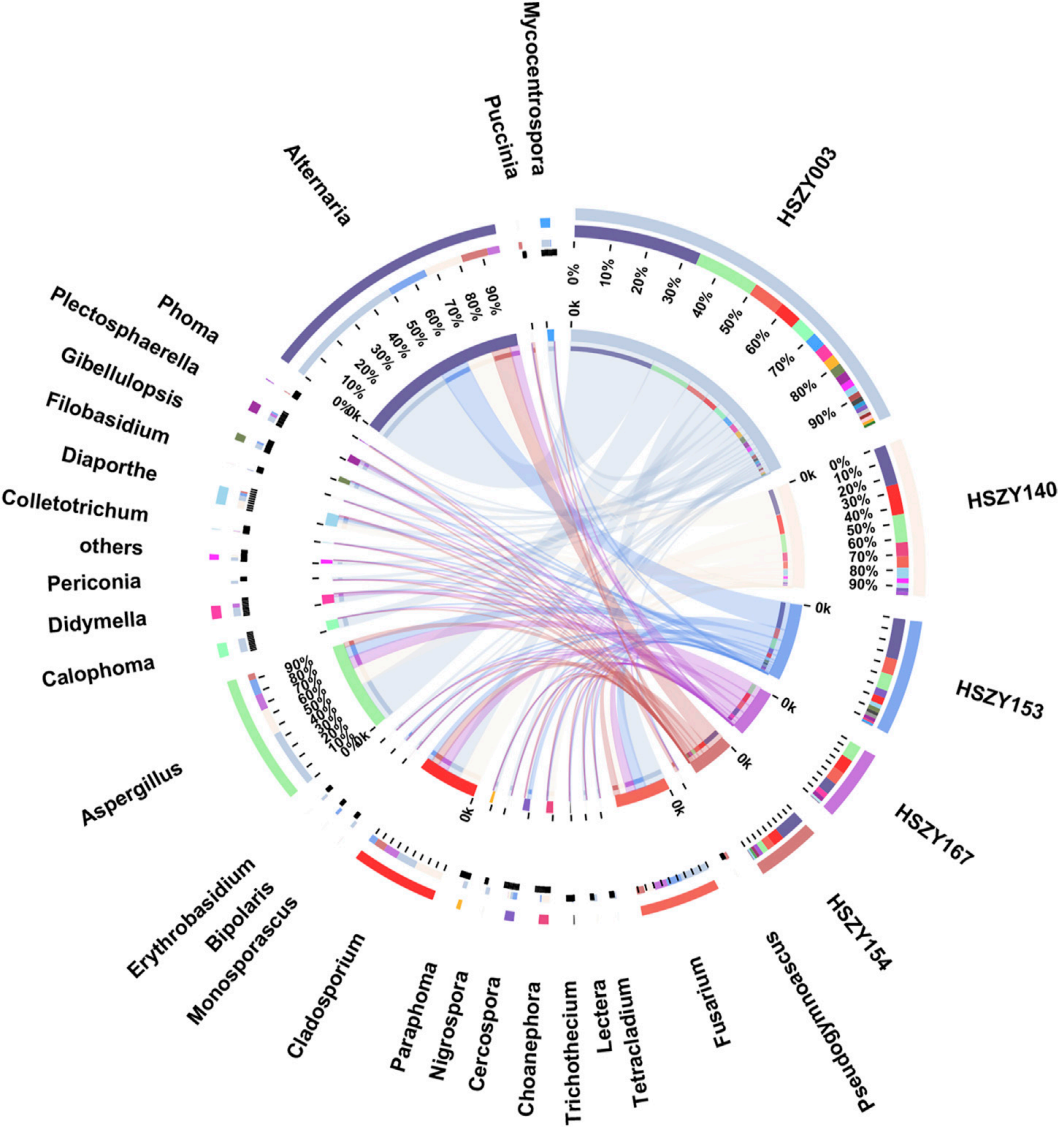
The fungal community distribution for each sample at the genus level. The data were visualized using Circos. The left half-circle indicates the distribution ratio of species in different samples at the genus level: the outer ribbon represents the species, the inner color of the ribbon represents different groups, and the length represents the proportion of the sample for a particular genus. The right half-circle indicates the species composition in each sample: the color of the outer ribbon represents samples from different groups, the color of the inner ribbon represents the composition of different species in each sample, and the length of the ribbon represents the relative abundance of the corresponding species.

This study analyzed the fungal communities in five FKDSW samples *via* ITS2. In total, 40 fungal ITS2 OTUs were obtained, and the BLAST results indicated that a total of 17 families that included 26 genera of fungal species were detected in all the FKDSW samples (Supplementary Table S9). Pleosporaceae represented the most abundant family, accounting for 17.54–41.29% of the fungal reads in five FKDSW samples, followed by Aspergillaceae, Cladosporiaceae, and Nectriaceae, as shown in Figure 3. Further taxonomical classification at the genus level demonstrated that *Alternaria*, *Aspergillus*, *Cladosporium,* and *Fusarium* were the most dominant genera among the 27 detected genera, with a relative abundance of 17.54–41.29%, 9.68–23.39%, 6.05–21.64%, and 7.56–19.30%, respectively, as shown in Figure 4. There were differences in fungal communities between the lab-made mock and four commercial FKDSW samples. *Alternaria* was the dominant genera in the four commercial samples, while *Aspergillus* was the most abundant genera in the lab-made mock sample. In addition, the fungal communities in the HSZY003, HSZY140, HSZY153, and HSZY154 commercial samples were composed of 21, 10, 12, and 8 genera, respectively, while the lab-made mock sample only contained seven genera, namely, *Alternaria*, *Aspergillus*, *Cladosporium*, *Colletotrichum*, *Didymella*, *Fusarium,* and *Plectosphaerella*.

## Discussion

### Challenges and Biotechnical Advances of Shotgun Metabarcoding in Traditional Herbal Product Authentication

Shotgun metagenomics is widely used for the detection and characterization of microbial community structures and functions, including those found in the human intestinal tract (Laudadio et al., 2018), in soil (Khodakova et al., 2014), in food (Yang et al., 2016), and in marine water (Venter et al., 2004), as well as terrestrial arthropod communities (Zhou et al., 2013). Xin et al. (2018a) applied shotgun metagenomic sequencing for the first time to detect the biological ingredients in Longdan Xiegan Wan, verifying the feasibility of the method for monitoring the species composition in traditional herbal patent medicine. A significant difference was evident compared with Longdan Xiegan Wan since the content of the highest dosage of the all the herbal materials exceeded that of the lowest dosage in FKDSW more than 16 times. In this study, shotgun metabarcoding was performed directly to analyze the biological ingredients in lab-made mock and commercial FKDSW samples, while the raw data obtained were qualified for subsequent data processing, such as assembly, annotation, and alignment.

Thereafter, all the assembly sequences of the ITS2, *matK*, and *rbcL* barcodes were successfully obtained for the six prescribed herbal ingredients in the mock FKDSW sample. The obtained assembly sequences were compared with each reference sequence, and the results indicated that all the assembly sequences were identical to the corresponding reference sequences except for part of the ITS2 and *matK* assembly sequences denoting *Paeonia lactiflora*, *Saussurea costus,* and *Bupleurum chinense*. A possible reason for the base differences between the assembly sequences and the reference sequences may be the multiple ITS2 copies or individual differences among plants (Álvarez, 2003). Although there are frequently hundreds of ITS2 copies with potentially dozens of different sequences in each genome, all ITS2 variants are sufficient for species identification in most cases (Song et al., 2012). Furthermore, there are positive and significant correlations between the amount of content of each herbal material and the read depth of ITS2, *matK*, *rbcL* barcodes (Zhou et al., 2013; Bista et al., 2018), however, the change ratio of the read depth with the amount of content is not with a good linear relationship. Finally, the analytical results suggested that all the prescribed and non-prescribed ingredients in the four commercial samples were detected based on the combination of three DNA barcodes even at low level amount of content. This verified that shotgun metabarcoding can overcome the biased PCR amplification and is particularly suitable for authenticating the biological ingredients in traditional herbal patent medicine with distinct variances in the herbal material dosages.

A total of 51 species were identified at the species level based on the ITS2 sequences that were obtained, which included all the prescribed medicinal materials and most of the exogenous biological contamination. Nevertheless, only nine species were determined at the species level based on the *matK* and *rbcL* sequences. Some Asteraceae and Apiaceae species were resolved at the genera level or higher, such as the original plant species of Angelicae Sinensis Radix (Danggui) and Bupleuri Radix (Chaihu). For this phenomenon, it may be caused by the identification efficiency of different DNA barcodes (Gao et al., 2010; China Plant et al., 2011). In 2010, Chen et al. tested the performance of seven candidate DNA barcodes (*psbA-trnH*, *matK*, *rbcL*, *rpoC1*, *ycf5*, ITS2, and ITS) from medicinal plants. The results showed that the identification efficiency of the ITS2 region at the species level was 92.7%, and it was proposed as the standard DNA barcoding for medicinal plants (Chen et al., 2010). Compared to the ITS/ITS2 regions, the identification efficiency of the *matK* and *rbcL* DNA barcodes was too low to distinguish more species in Asteraceae and Apiaceae (Gao et al., 2010; Liu et al., 2014). Although the chloroplast gene in *matK* and *rbcL* did not recognize the species in some cases, they displayed an excellent ability to identify orchids (Lahaye et al., 2008) or discriminate among species in congeneric pair-wise comparisons (Newmaster et al., 2006). Moreover, chloroplast genomes have the advantage of maternal inheritance, avoiding genetic recombination, and the high-copy number of plastids per cell are accessible to extract genomic DNA (Twyford and Ness, 2017). Therefore, we recommend that *matK* and *rbcL* as the complementary barcodes to ITS2 for the identification of medicinal plants. To sum up, this study established a biological method for monitoring traditional herbal patent medicine *via* shotgun metabarcoding with ITS2 as the core barcode, and *matK* and *rbcL* as the supplementary barcodes, effectively supplementing traditional identification methods.

### Shotgun Metabarcoding is a “Best Practice” Method for the Detection of Biological Contamination Like Non-Authentic Ingredient, Weed, and Fungi Species

In addition to the prescribed herbal medicines, this study identified some unlabeled biological species, such as *Bupleurum falcatum*, weeds, and several fungi species. Bupleuri Radix was officially derived from the dried root of the Asteraceae plants, *Bupleurum chinense,* and *Bupleurum scorzonerifolium* Willd., according to the Chinese Pharmacopeia (Commission, 2020). However, there are still more than 20 additional species of the genus *Bupleurum* that are habitually utilized in China as a non-authentic ingredient for Bupleuri Radix (Liu et al., 2011). *Bupleurum falcatum* was introduced from Japan into China in the early 1970s and has been recorded as the legal original species of Bupleuri Radix in Japanese and Korean Pharmacopoeia (Yuan et al., 2017; Yeom et al., 2018). In 2007, Zhu et al. (2017) analyzed the chemical profiles of *Bupleurum chinense*, *Bupleurum yinchowense* R.H.Shan & Y.Li, and *Bupleurum falcatum* using UHPLC-QTOF-MS. The results indicated that the chemical profiles of the root samples from the three *Bupleurum* species were similar, especially the characteristic saikosaponins. However, a study suggested that the extracts of *Bupleurum chinense*, *Bupleurum falcatum,* and *Bupleurum scorzonerifolium* exhibit different pharmacological activities (Yuan et al., 2017). Using the FKDSW with the dried root of *Bupleurum falcatum* instead of the root of *Bupleurum chinense* or *Bupleurum scorzonerifolium* poses a potential threat to its clinical efficacy.

A total of 10 plant families with 16 genera that included 25 weed species were detected in five FKDSW samples, such as species from *Artemisia* (*Artemisia annua*), *Humulus* (*Humulus scandens*), and *Ipomoea* (*Ipomoea purpurea*), which are all common field weeds found on the farmland where traditional herbal materials are cultivated (Ya-ling et al., 2018). It is not unusual for weed species to be mixed into traditional herbal products (Xin et al., 2018b). Jia et al. (2017) detected a variety of non-listed plant species in the commercial Yimu Wan samples using single-molecule realtime sequencing and DNA barcoding. Of these, species from *Humulus*, *Ipomoea*, *Artemisia,* and *Amaranthus* are mostly denoted common weeds, and sequences belonging to these species accounted for a large proportion of the total sequences. Controlling field weeds is not only an essential factor that impacts the artificial standardized cultivation of traditional herbal materials but is also a significant problem affecting global agricultural production. In addition, there are strict standards about using pesticides and fertilizers during the cultivation process of traditional herbal materials (Sang, 2017). Therefore, weed control is challenging during the cultivation of traditional herbal materials because of strict requirements, high standards, and high labor costs. “Leonuri Herba (Yimucao)” is the dried ground part of *Leonurus japonicas*. And it easily mixes with the leaves of some *Artemisia* plants during the manufacturing process due to their overlapping morphological features. *Ipomoea purpurea*/*Ipomoea nil,* and *Humulus scandens* are twining herbs (Zhengyi et al., 2013) that twine around the stem of *Leonurus japonicus* while growing. This might be the reason why *Artemisia*, *Humulus,* and *Ipomoea* were the most common plant genera found in five FKDSW samples.

Similar to agricultural products, plants cultivated for herbal medicines are also vulnerable to fungal contamination during the process of cultivation, processing, transportation, and storage (Xing et al., 2016; Guo et al., 2020; He et al., 2020). This study identified a total of 17 families that included 26 genera of fungal species in five FKDSW samples, and there is a certain difference in fungal communities between the mock and the commercial FKDSW products. The reason for this difference in fungal communities may be due to that the herbal materials for making the mock sample were purchased from medicinal stores, while the raw herbal materials of the commercial products were collected by the FKDSW manufacturers (He et al., 2020). Furthermore, most of the detected fungi species were common pathogenic fungi typically present during the growth period of field plants. For example, *Cercospora canescens* is an important pathogen of Cercospora leaf spot that can lead to serious yield loss of Yardlong bean (Duangsong et al., 2018). It is a remarkable fact that the *Alternaria*, *Aspergillus,* and *Fusarium* genera represent the most common infective fungi in agricultural products, food, and herbal medicines among the dominant bacteria detected, and are potential mycotoxin-producing microbial flora (Xing et al., 2016; Alshannaq and Yu, 2017; Guo et al., 2020). Of these, aflatoxins (AFs) and ochratoxin A, produced by *Aspergillus,* denote the most important contaminants due to their strong carcinogenicity (Perrone and Gallo, 2017). Therefore, the 2020 edition of the Chinese Pharmacopoeia (Commission, 2020) expressly limit the content of AFs in Chinese medicinal materials, such as Jujubae Fructus (Dazao), Cassiae Semen (Juemingzi), and Hordei Fructus Germinatus (Maiya), which are moldy-prone Chinese medicinal materials, expressed as aflatoxin B_1_ (AFB_1_)≤ 5 μg/kg, and the total amount of aflatoxin G_1_, aflatoxin G_2_, AFB_1_ and aflatoxin B_2_ ≤ 10 μg/kg. Consequently, it is suggested that the quality control of the entire industrial chain should be reinforced using DNA barcoding technology during the cultivation, harvesting, processing, transportation, and storage processes of herbal medicines to ensure the safety and efficacy of traditional herbal medicines.

## Data Availability

The datasets presented in this study can be found in online repositories. The names of the repository/repositories and accession number(s) can be found below: NCBI Bioproject: PRJNA663116, BioSample: SAMN16132426, SAMN16132427, SAMN16132428, SAMN16132429, SAMN16132430. SRA accession numbers are: SRR12640731, SRR12640730, SRR12640729, SRR12640728, SRR12640727. The DNA barcoding sequences assembled from the Sanger sequencing datasets presented in this study can be found in the NCBI GenBank online repository. The accession numbers for these DNA barcoding sequences are MN712234, MN712235, MN712236, MN712237, MN712238, MN712239, MN746764, MW000342, MW000343, MW000344, MN729559, MW000336, MW000337.
