# Supplementary figures and images for: Biological Ingredient Analysis of Traditional Herbal Patent Medicine Fuke Desheng Wan Using the Shotgun Metabarcoding Approach
Source: Front Pharmacol. 2021 Aug 17;12:607197. doi: 10.3389/fphar.2021.607197 (PMC8416078; doi:10.3389/fphar.2021.607197)

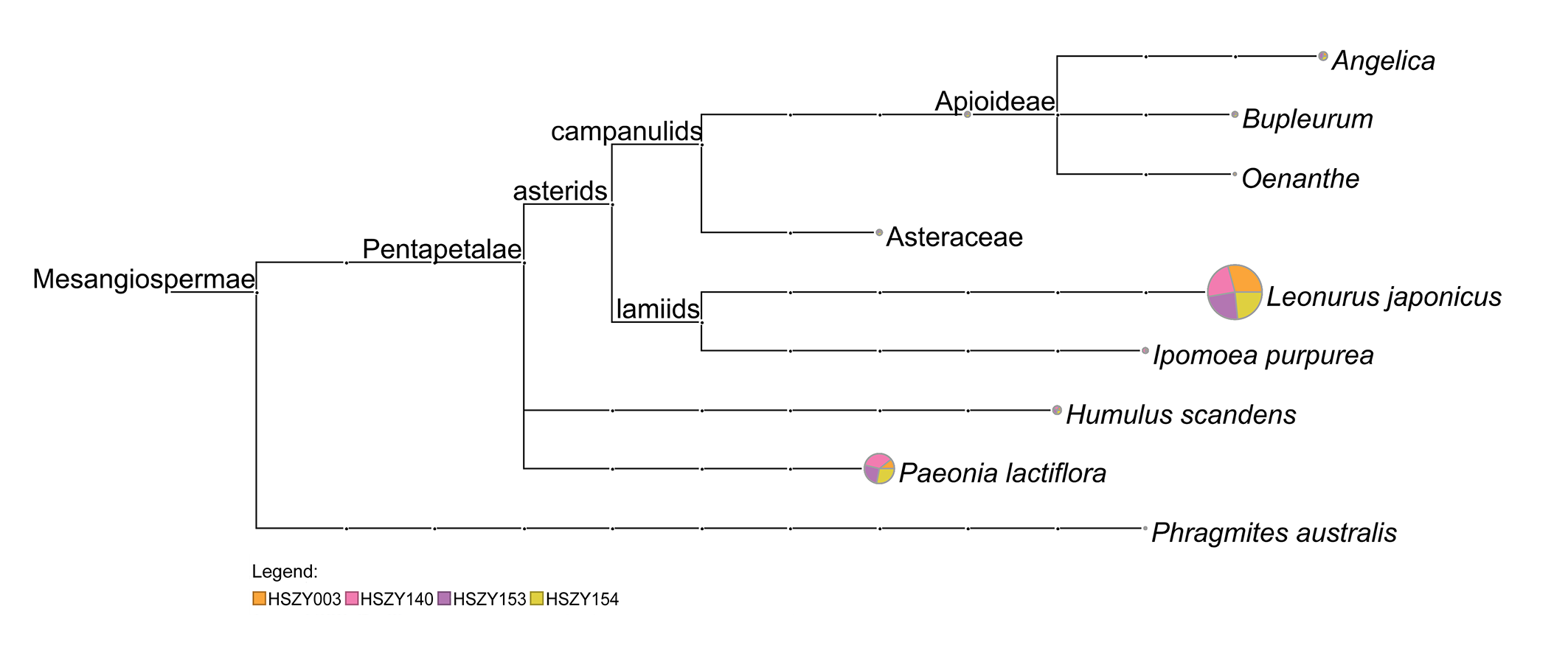

Supplement: Supplementary file 1 [file Image3.TIF]

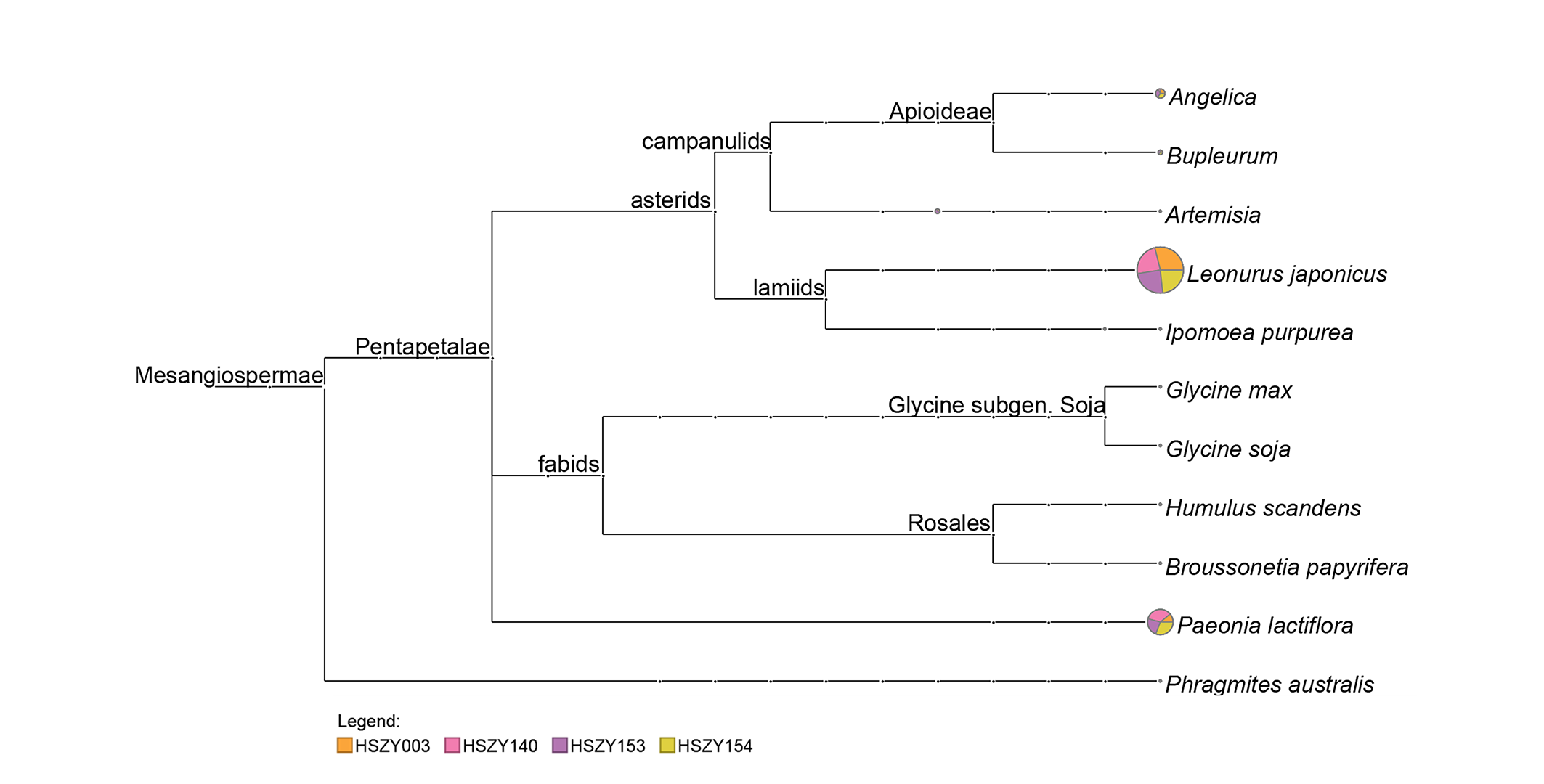

Supplement: Supplementary file 2 [file Image2.TIF]

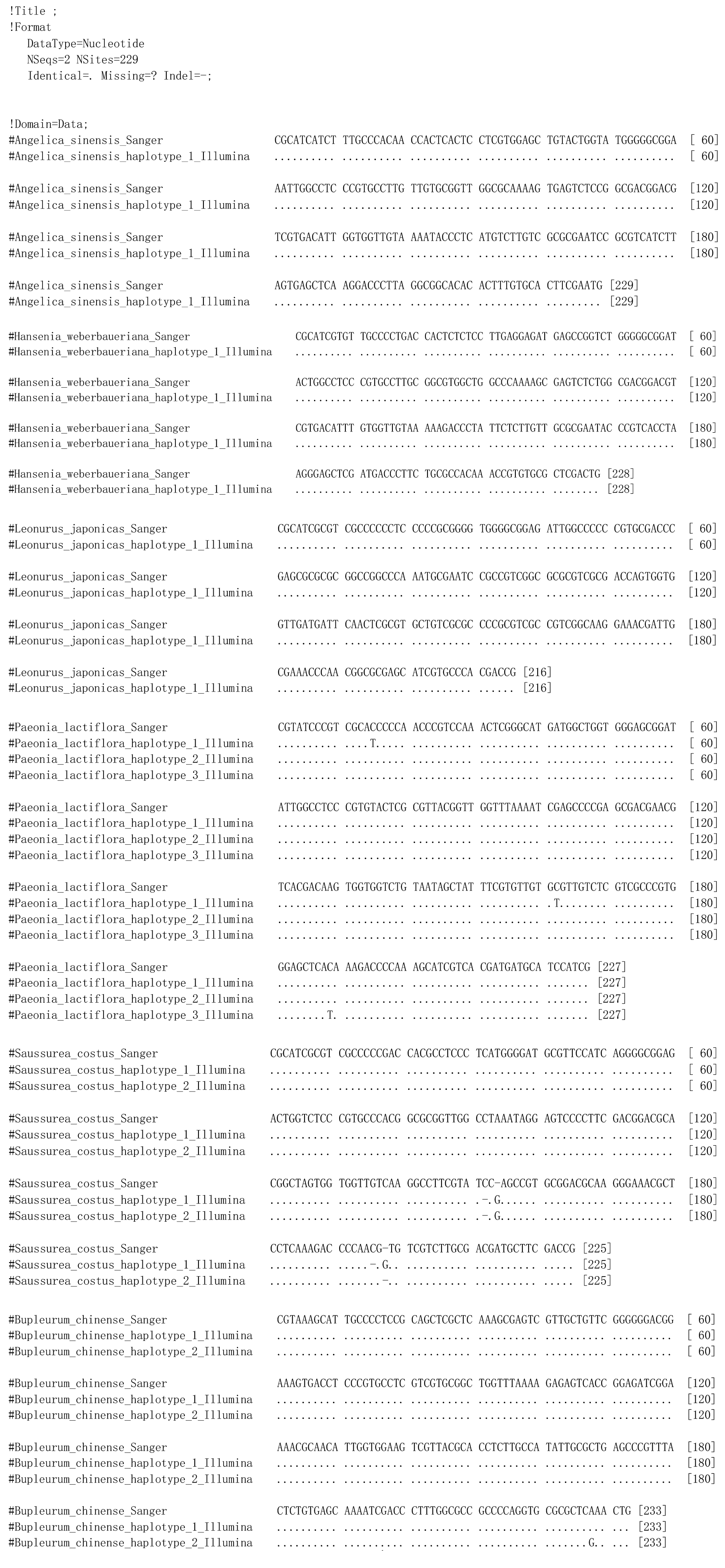

Supplement: Supplementary file 3 [file Image1.TIF]
