# Supplementary material for: Biological Ingredient Analysis of Traditional Herbal Patent Medicine Fuke Desheng Wan Using the Shotgun Metabarcoding Approach
Source: Front Pharmacol. 2021 Aug 17;12:607197. doi: 10.3389/fphar.2021.607197 (PMC8416078; doi:10.3389/fphar.2021.607197)
Supplement: Supplementary file 4 [file DataSheet1.docx]

Supplementary Materials

# Supplementary Tables

**Supplementary Table 1.** The sample information of the herbal materials in the lab-made mock FKDSW sample.

| Herbal materials | Original species | Medicinal part | Processing method | The location of the drug store |
| --- | --- | --- | --- | --- |
| Angelicae Sinensis Radix (Danggui) | *Angelica sinensis* | Dried roots | Cleaning and roasting | Beijing |
| Paeoniae Radix Alba (Baishao) | *Paeonia lactiflora* | Dried roots | Cleaning, steaming and sun drying | Beijing |
| Aucklandiae Radix (Muxiang) | *Saussurea costus* | Dried roots | Cleaning, cutting into segments or slices, drying and striking | Beijing |
| Notopterygii Rhizoma et Radix (Qianghuo) | *Hansenia weberbaueriana* | Dried roots and rhizomes | Cleaning and sun drying | Beijing |
| Leonuri Herba (Yimucao) | *Leonurus japonicus* | Fresh or dried aboveground parts | Cutting into segments or slices and sun drying | Beijing |
| Bupleuri Radix (Chaihu) | *Bupleurum chinense* | Dried roots | Cleaning and drying | Beijing |

**Supplementary Table 2.** The quantity and quality of the DNA extracted from the six herbal ingredients in FKDSW.

| Sample ID | Herbal material Latin name (pinyin name) | Concentration（ng/μL） | A_260_/A_280_ |
| --- | --- | --- | --- |
| HSYC2002 | Angelicae Sinensis Radix (Danggui) | 377.4 | 2.1 |
| HSYC2036 | Paeoniae Radix Alba (Baishao) | 298.2 | 1.7 |
| HSYC2037 | Aucklandiae Radix (Muxiang) | 251.1 | 2.0 |
| HSYC2038 | Notopterygii Rhizoma et Radix (Qianghuo) | 139.5 | 1.7 |
| HSYC2039 | Leonuri Herba (Yimucao) | 166.5 | 1.4 |
| HSYC2078 | Bupleuri Radix (Chaihu) | 721 | 1.9 |

**Supplementary Table 3.** The quantity and quality of the DNA extracted from the five FKDSW samples.

| Sample ID | Formulation | Concentration (ng/μL） | A_260_/A_280_ |
| --- | --- | --- | --- |
| HSZY003 | honeyed pills | 148.6 | 1.9 |
| HSZY140 | honeyed pills | 91.8 | 1.9 |
| HSZY153 | honeyed pills | 91.7 | 1.8 |
| HSZY154 | honeyed pills | 135.8 | 1.8 |
| HSZY167 | honeyed pills | 84.7 | 1.9 |

**Supplementary Table 4.** A summary of the high-throughput sequencing data and the number of reads enriched for ITS2, *matK*, and *rbcL.*

| Sample ID | Bases (Gb) | Total reads | The number of enriched reads for each DNA barcode | | | |
| --- | --- | --- | --- | --- | --- | --- |
|  |  |  | ITS2 | *matK* | *rbcL* | Total |
| HSZY003 | 18.14 | 60479906 | 62265 | 25668 | 49052 | 136985 |
| HSZY140 | 6.6 | 21999270 | 33897 | 2408 | 4933 | 41238 |
| HSZY153 | 8.43 | 28083999 | 39188 | 3679 | 7208 | 50075 |
| HSZY154 | 8.19 | 27285055 | 36372 | 3744 | 7260 | 47376 |
| HSZY167 | 10.8 | 36008506 | 45363 | 17143 | 33373 | 95879 |

**Supplementary Table 5.** The authentic and non-authentic ingredients have been detected in four commercial FKDSW samples using shotgun metabarcoding.

| Expected ingredients | Expected ingredient species | Detected ingredient species | | | |
| --- | --- | --- | --- | --- | --- |
|  |  | HSZY003 | HSZY140 | HSZY153 | HSZY154 |
| Angelicae Sinensis Radix (Danggui) | *Angelica sinensis* | *A. sinensis* | *A. sinensis* | *A. sinensis* | *A. sinensis* |
| Paeoniae Radix Alba (Baishao) | *Paeonia lactiflora* | *P. lactiflora* | *P. lactiflora* | *P. lactiflora* | *P. lactiflora* |
| Aucklandiae Radix (Muxiang) | *Saussurea costus* | *S. costus* | *S. costus* | *S. costus* | *S. costus* |
| Notopterygii Rhizoma et Radix (Qianghuo) | *Hansenia weberbaueriana or*  *Hansenia forbesii* | *H. weberbaueriana and H. forbesii* | *H. weberbaueriana* | *H. weberbaueriana and H. forbesii* | *H. weberbaueriana and H. forbesii* |
| Leonuri Herba (Yimucao) | *Leonurus japonicus* | *L. japonicus* | *L. japonicus* | *L. japonicus* | *L. japonicus* |
| Bupleuri Radix (Chaihu) | *Bupleurum chinense or*  *Bupleurum scorzonerifolium* | *B. chinense* | *B. chinense* | *B. chinense* | *B. chinense* |
| non-authentic ingredients | *Bupleurum falcatum* | *B. falcatum* | *B. falcatum* | *B. falcatum* | *B. falcatum* |

**Supplementary Table 6.** The number of paired-end reads of the contaminating plant species detected in five FKDSW samples via shotgun sequencing based on the ITS2 DNA barcoding region.

| Family | Genus | Species | HSZY003 | HSZY140 | HSZY153 | HSZY154 | HSZY167 |
| --- | --- | --- | --- | --- | --- | --- | --- |
| Plantaginaceae | *Plantago* | *Plantago asiatica* L. | 11 | 0 | 0 | 0 | 0 |
| Lamiaceae | *Panzerina* | *Panzerina lanata* (L.) Soják | 88 | 79 | 93 | 65 | 0 |
| Fabaceae | *Astragalus* | *Astragalus mongholicus* Bunge | 10 | 0 | 0 | 0 | 0 |
|  | *Glycine* | *Glycine max* (L.) Merr. | 180 | 19 | 198 | 78 | 0 |
|  | *Robinia* | *Robinia pseudoacacia* L. | 112 | 8 | 10 | 0 | 0 |
| Poaceae | *Phragmites* | *Phragmites australis* (Cav.) Trin. ex Steud. | 33 | 17 | 9 | 0 | 0 |
|  | *Setaria* | *Setaria viridis* (L.) P.Beauv. | 64 | 7 | 0 | 9 | 0 |
|  | *Oryza* | *Oryza sativa* L. | 162 | 0 | 0 | 0 | 0 |
| Malvaceae | *Abutilon* | *Abutilon theophrasti* Medik. | 43 | 38 | 11 | 11 | 0 |
|  | *Hibiscus* | *Hibiscus trionum* L. | 0 | 0 | 0 | 85 | 0 |
| Asteraceae | *Eschenbachia* | *Erigeron canadensis* L. | 40 | 8 | 29 | 45 | 26 |
|  |  | *Erigeron sumatrensis* Retz. | 27 | 0 | 0 | 21 | 0 |
|  | *Aster* | *Aster pekinensis* (Hance) F.H.Chen | 68 | 8 | 30 | 44 | 0 |
|  | *Bidens* | *Bidens bipinnata* L. | 24 | 0 | 9 | 0 | 0 |
|  | *Artemisia* | *Artemisia annua* L. | 654 | 71 | 315 | 447 | 47 |
|  |  | *Artemisia indica* Willd. | 121 | 0 | 45 | 52 | 14 |
|  |  | *Artemisia argyi*H.Lév. & Vaniot | 141 | 0 | 57 | 64 | 0 |
|  |  | *Artemisia stolonifera* (Maxim.) Kom. | 19 | 0 | 0 | 7 | 0 |
|  |  | *Artemisia vulgaris* L. | 179 | 15 | 89 | 89 | 0 |
|  |  | *Artemisia scoparia* Waldst. & Kit. | 24 | 22 | 122 | 46 | 51 |
|  |  | *Artemisia sieversiana* Ehrh. ex Willd. | 19 | 0 | 19 | 55 | 78 |
|  |  | *Artemisia sp.* | 509 | 38 | 293 | 258 | 127 |
| Bignoniaceae | *Incarvillea* | *Incarvillea sinensis* Lam. | 8 | 0 | 48 | 20 | 0 |
| Ulmaceae | *Ulmus* | *Ulmus sp.* | 66 | 35 | 163 | 170 | 0 |
| Cannabaceae | *Cannabis* | *Cannabis sativa* L. | 48 | 0 | 33 | 43 | 0 |
|  | *Humulus* | *Humulus scandens* (Lour.) Merr. | 1045 | 562 | 568 | 604 | 81 |
| Brassicaceae | *Brassica* | *Brassica sp.* | 114 | 0 | 48 | 47 | 0 |
| Convolvulaceae | *Cuscuta* | *Cuscuta australis* Yunck. | 40 | 0 | 0 | 6 | 0 |
|  | *Ipomoea* | *Ipomoea nil* (L.) Roth | 54 | 51 | 64 | 60 | 65 |
|  |  | *Ipomoea purpurea* (L.) Roth | 275 | 342 | 630 | 164 | 110 |
| Salicaceae | *Populus* | *Populus sp.* | 9 | 0 | 19 | 55 | 0 |
|  |  | *Populus deltoides* W.Bartram ex Marshall | 13 | 0 | 31 | 34 | 0 |

**Supplementary Table 7.** The number of paired-end reads of the contaminating plant species detected in five FKDSW samples via shotgun sequencing based on the *matK* DNA barcoding region.

| Family | Genus | Species | HSZY003 | HSZY140 | HSZY153 | HSZY154 | HSZY167 |
| --- | --- | --- | --- | --- | --- | --- | --- |
| Poaceae | *Phragmites* | *Phragmites australis* (Cav.) Trin. ex Steud. | 222 | 0 | 0 | 18 | 0 |
| Convolvulaceae | *Ipomoea* | *Ipomoea purpurea* (L.) Roth | 87 | 0 | 0 | 0 | 23 |
|  |  | others | 99 | 0 | 0 | 0 | 61 |
| Fabaceae | *Glycine* | *Glycine soja* Siebold & Zucc. | 28 | 0 | 0 | 0 | 0 |
|  |  | *Glycine max* | 23 | 0 | 0 | 0 | 0 |
| Cannabaceae | *Humulus* | *Humulus scandens* (Lour.) Merr. | 191 | 21 | 20 | 0 | 0 |
| Moraceae | *Broussonetia* | *Broussonetia papyrifera* (L.) L'Hér. ex Vent. | 141 | 0 | 53 | 0 | 0 |
| Asteraceae | *Artemisia* | others | 136 | 0 | 0 | 0 | 26 |

**Supplementary Table 8.** The number of paired-end reads of the contaminating plant species detected in five FKDSW samples via shotgun sequencing based on the *rbcL* DNA barcoding region.

| Family | Genus | Species | HSZY003 | HSZY140 | HSZY153 | HSZY154 | HSZY167 |
| --- | --- | --- | --- | --- | --- | --- | --- |
| Convolvulaceae | *Ipomoea* | *Ipomoea purpurea* (L.) Roth | 144 | 40 | 21 | 0 | 28 |
|  |  | others | 0 | 0 | 0 | 0 | 28 |
| Poaceae | *Phragmites* | *Phragmites australis* (Cav.) Trin. ex Steud. | 236 | 0 | 0 | 21 | 0 |
| Cannabaceae | *Humulus* | *Humulus scandens* (Lour.) Merr. | 292 | 44 | 76 | 69 | 23 |
| Apiaceae | *Oenanthe* | others | 111 | 0 | 0 | 0 | 0 |
|  | others |  | 198 | 0 | 34 | 47 | 299 |

**Supplementary Table 9.** The number of paired-end reads of the fungal species detected in five FKDSW samples via shotgun sequencing based on the ITS2 DNA barcoding region.

| Family | Genus | Species | HSZY003 | HSZY140 | HSZY153 | HSZY154 | HSZY167 |
| --- | --- | --- | --- | --- | --- | --- | --- |
| Pleosporaceae | *Alternaria* | *Alternaria alternata* | 294 | 94 | 97 | 64 | 30 |
|  | *Bipolaris* | *Bipolaris zeicola* | 0 | 0 | 8 | 0 | 0 |
| Aspergillaceae | *Aspergillus* | *Aspergillus flavus* | 80 | 6 | 23 | 15 | 0 |
|  |  | *Aspergillus niger* | 12 | 0 | 0 | 0 | 0 |
|  |  | *Aspergillus sp.* | 53 | 60 | 16 | 0 | 40 |
| Didymellaceae | *Calophoma* | *Calophoma complanata* | 47 | 0 | 0 | 0 | 0 |
| Mycosphaerellaceae | *Cercospora* | *Cercospora canescens* | 10 | 9 | 6 | 0 | 0 |
|  |  | *Cercospora sp.* | 0 | 0 | 12 | 0 | 0 |
|  | *Mycocentrospora* | *Mycocentrospora acerina* | 33 | 0 | 5 | 0 | 0 |
| Choanephoraceae | *Choanephora* | *Choanephora infundibulifera* | 0 | 24 | 0 | 0 | 0 |
|  |  | *Choanephora cucurbitarum* | 0 | 9 | 5 | 0 | 0 |
| Cladosporiaceae | *Cladosporium* | *Cladosporium sp.* | 52 | 72 | 18 | 28 | 37 |
| Glomerellaceae | *Colletotrichum* | *Colletotrichum gloeosporioides* | 0 | 11 | 0 | 0 | 7 |
| Diaporthaceae | *Diaporthe* | *Diaporthe sp.* | 16 | 25 | 10 | 5 | 0 |
| Didymellaceae | *Didymella* | *Didymella sp.* | 31 | 0 | 0 | 0 | 14 |
|  | *Phoma* | *Phoma sp.* | 0 | 8 | 0 | 10 | 0 |
|  | others | *Didymellaceae sp.* | 17 | 12 | 0 | 0 | 0 |
| Filobasidiaceae | *Filobasidium* | *Filobasidium magnum* | 13 | 0 | 0 | 0 | 0 |
| Nectriaceae | *Fusarium* | *Fusarium sp.* | 74 | 27 | 39 | 20 | 33 |
| Plectosphaerellaceae | *Gibellulopsis* | *Gibellulopsis sp.* | 22 | 0 | 10 | 0 | 0 |
|  | *Plectosphaerella* | *Plectosphaerella cucumerina* | 9 | 0 | 0 | 0 | 10 |
|  |  | *Plectosphaerella sp.* | 12 | 0 | 8 | 0 | 0 |
|  | *Lectera* | *Lectera sp.* | 7 | 0 | 0 | 0 | 0 |
| Trichosphaeriaceae | *Nigrospora* | *Nigrospora sp.* | 6 | 0 | 0 | 0 | 0 |
| Phaeosphaeriaceae | *Paraphoma* | *Paraphoma sp.* | 25 | 0 | 0 | 0 | 0 |
| Periconiaceae | *Periconia* | *Periconia sp.* | 8 | 0 | 0 | 0 | 0 |
| Pseudeurotiaceae | *Pseudogymnoascus* | *Pseudogymnoascus sp.* | 0 | 0 | 0 | 6 | 0 |
| Pucciniaceae | *Puccinia* | *Puccinia chrysanthemi* | 0 | 0 | 0 | 7 | 0 |
| Erythrobasidiaceae | *Erythrobasidium* | *Erythrobasidium* | 9 | 0 | 0 | 0 | 0 |
| no rank | *Tetracladium* | *Tetracladium sp.* | 10 | 0 | 0 | 0 | 0 |
| no rank | *Monosporascus* | *Monosporascus sp.* | 8 | 0 | 0 | 0 | 0 |
| no rank | *Trichothecium* | *Trichothecium roseum* | 11 | 0 | 8 | 0 | 0 |
